# Formulating a Community-Centric Indicator Framework to Quantify One Health Drivers of Antibiotic Resistance: A Preliminary Step towards Fostering ‘Antibiotic-Smart Communities’

**DOI:** 10.3390/antibiotics13010063

**Published:** 2024-01-08

**Authors:** Philip Mathew, Sujith J. Chandy, Satya Sivaraman, Jaya Ranjalkar, Hyfa Mohammed Ali, Shruthi Anna Thomas

**Affiliations:** 1 ReAct Asia Pacific, Department of Pharmacology and Clinical Pharmacology, Christian Medical College, Vellore 632002, Tamil Nadu, India; philipmathewrap@gmail.com (P.M.); satyasagar@gmail.com (S.S.); hyfarap@gmail.com (H.M.A.); shruthiannarap@gmail.com (S.A.T.); 2 Department of Pharmacology and Clinical Pharmacology, Christian Medical College, Vellore 632002, Tamil Nadu, India; sjchandy@cmcvellore.ac.in

**Keywords:** antimicrobial resistance, National Action Plans, AMR, WASH, IPC, One Health, ASC

## Abstract

Antibiotic resistance (ABR) is increasing the mortality and morbidity associated with infectious diseases, besides increasing the cost of healthcare, saturating health system capacity, and adversely affecting food security. Framing an appropriate narrative and engaging local communities through the ‘One Health’ approach is essential to complement top-down measures. However, the absence of objective criteria to measure the performance of ABR interventions in community settings makes it difficult to mobilize interest and investment for such interventions. An exercise was therefore carried out to develop an indicator framework for this purpose. A comprehensive list of indicators was developed from experiences gathered through community engagement work in a local *panchayat* (small administrative area) in Kerala, India and a consultative process with health, veterinary, environment, and development experts. A prioritization exercise was carried out by global experts on ABR, looking at appropriateness, feasibility, and validity. A 15-point indicator framework was designed based on the prioritization process. The final set of indicators covers human health, animal health, environment management, and Water Sanitation and Hygiene (WASH) domains. The indicator framework was piloted in the *panchayat* (located in Kerala), which attained a score of 34 (maximum 45). The score increased when interventions were implemented to mitigate the ABR drives, indicating that the framework is sensitive to change. The indicator framework was tested in four sites from three other Indian states with different socioeconomic and health profiles, yielding different scores. Those collecting the field data were able to use the framework with minimal training. It is hoped that, this indicator framework can help policymakers broadly understand the factors contributing to ABR and measure the performance of interventions they choose to implement in the community as part of National Action Plan on AMR.

## 1. Introduction

Antibiotic resistance (ABR) was associated with 4.95 million deaths and was the attributable cause of 1.27 million deaths in 2019 [1]. This is much higher than the previous estimate of 700,000 deaths per year [2]. The projected cost of ABR is also high, with the World Bank estimating a 1.1% loss in the global Gross Domestic Product (GDP) by 2050 and an annual reduction of USD 1 trillion per year beyond 2030 in the best-case scenario [3]. The burden of ABR is expected to be much higher in Lower–Middle-Income Countries (LMICs) due to their dysfunctional health systems, poor agricultural production practices, and sub-optimal environmental management [4]. Additionally, antibiotic consumption is increasing rapidly in many LMICs, thereby increasing ABR [5]. Therefore, action to contain ABR should be a priority for the public health system, especially in low-resource settings.

The global efforts made to tackle ABR have been anchored in the Global Action Plan on Antimicrobial Resistance (GAP-AMR) adopted by the World Health Assembly in 2015 [6]. Since then, most countries have adopted their own action plans, but very few of them have been funded and fully operationalized [7]. The Inter-Agency Coordination Group on AMR (IACG-AMR) submitted its report to the United Nations Secretary-General on a globally coordinated response and called for a systematic and meaningful engagement of all stakeholders at global, regional, national, and local levels. The report conveyed the need for contextualized interventions based on locally generated data and insights rather than on a uniform strategy [8]. Engaging local organizations and governance structures for broad-basing ABR containment efforts has been a consistent recommendation in several documents since the Jim O’Neill report was released. All of these documents also call for the engagement of communities in a meaningful and systematic manner [8]. Framing the right narrative for ABR at the ground level to engage local communities and creating a bottom-up process to supplement national and sub-national action plans have been challenging [9]. Studies have shown that there are also language and perceptional issues associated with ABR [10].

Recently, studies have shown that community-based interventions could be beneficial in reducing inappropriate antibiotic use [11]. Community engagement interventions could also facilitate ABR behavior change, specifically in LMICs, because they employ a contextualized approach that supports communities to develop locally relevant and viable solutions [12]. For successful community engagement in ABR it is important to understand the local context, develop relationships with key stakeholders, build motivation and trust, and engage with them on the topic of antibiotics and ABR [13].

While there are some examples of community engagement in ABR, our literature review did not yield any attempts to quantify ABR at the community level. It was therefore deemed important to conceptualize a community-centric indicator framework that could help policymakers (both nationally and locally), local government officials, and other relevant stakeholders to establish a baseline, understand the issues and factors contributing to ABR, as well as measure the impact of the interventions they choose to implement in that community. This paper is therefore a description of such a framework and the multi-stage process we undertook in its development, so that others may also be able to use this framework in similar low-resource settings.

In addition, the framework could also be used to aim for ‘antibiotic-smart communities’. Antibiotic smartness can be explained as the preparedness of a community to effectively and sustainably tackle ABR by addressing the drivers of ABR with a One Heath lens such as by taking measures to prevent infections, improve awareness, and promote the rational use of antibiotics.

## 2. Methods

ReAct is part of an independent science, policy, and advocacy-based network that has been working on antibiotic resistance since 2005. ReAct Asia Pacific (RAP) is one of the regional nodes of ReAct. RAP started working on the concept of an ‘antibiotic-smart communities’ with the hypothesis that the activities for ABR containment are predominantly at the national and subnational level, and community-level focus on ABR was inadequate. Developing an indicator framework was meant to help plug this gap.

**Figure 1:**
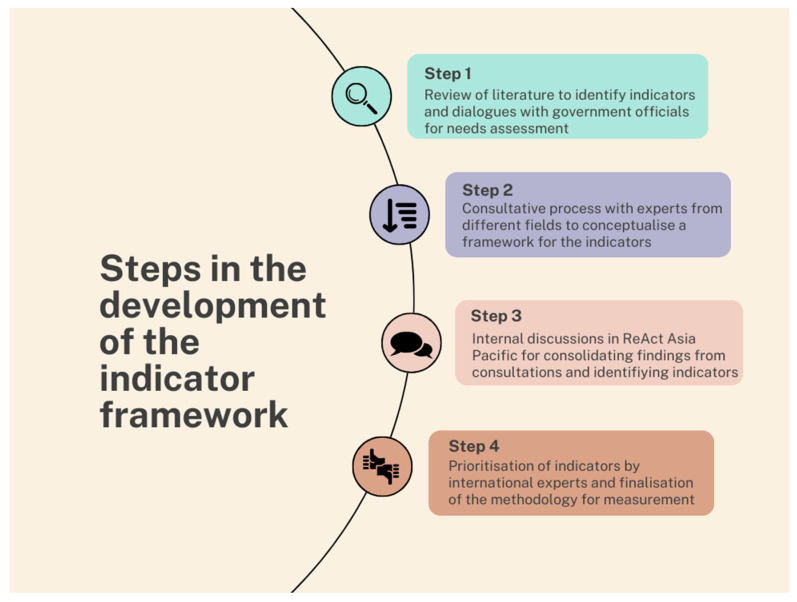
Steps in the process of the development of an indicator framework.

We selected Kerala as it was the first state to adopt a sub-national action plan on AMR. Kerala is an Indian state with high levels of literacy and education and a high human development index [14]. Kerala has a robust collectivist culture that fosters social cohesiveness and an ingroup aim [15,16]. In addition, Kerala’s strong local governance has engaged itself in managing and abating the impact of multiple health issues, including the provision of palliative care services and a decentralized response to COVID-19 rooted in the grassroots [17,18]. In this context, the investigators chose Kerala as the site to pilot the indicator framework since the setting is ideal for community engagement projects. Kerala’s state government is also supportive of community engagement initiatives given its history of community engagement [19,20]. This exploratory project was undertaken in a panchayat in the state of Kerala, India. A ‘panchayat’ is the smallest administrative unit in India’s three-tier local self-governance system, though the size and functions of a panchayat may vary widely between states. We selected Mallapuzhasserry, a panchayat with a population of 11,000 (as per the data from the last national census in India, conducted in 2011) and spread over a total area of 15 square kilometers. The project took place from 2018 to 2022. The steps in the project are summarized below in Figure 1.

Step 1: Literature review and needs assessment: As a first step, a literature review was undertaken in 2018 to identify existing frameworks. Dialogues were held with local government officials and other key stakeholders to identify their priorities concerning antimicrobial resistance. To gain access and build confidence, we used a healthcare delivery project managed by a local medical school and a community organization for piggybacking. These interactions gave an overview of ABR in the community and helped to draw a baseline narrative regarding existing efforts to combat ABR.

**Figure 2:**
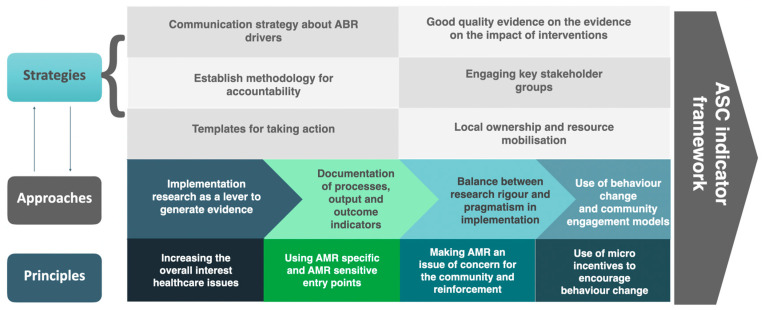
Conceptual framework used for developing the antibiotic-smart communities. Legend: ABR: Antibiotic Resistance; ASCs’: Antibiotic-Smart Communities; AMR: Antimicrobial Resistance.

Step 2: Meeting with experts from public/human health, animal health, environment, and agriculture: After the literature review, three consultation meetings were held in 2019, with experts from different sectors to conceptualize a framework for assessing different ABR drivers and their components. The experts deliberated on the need for a framework, what a hypothetical framework should contain, and possible principles that such a framework should entail to support the bottom-up approach for the development and implementation of state and national action plans. SDG indicators were used as a starting point for such discussions. The experts suggested that the framework should reflect drivers from ABR-specific and ABR-sensitive areas and capture the deficiencies in the system that influence these drivers. Figure 2 shows the conceptual framework used for developing the antibiotic-smart communities.

Step 3: Following the consultative meetings, the findings were consolidated and discussed internally (within ReAct Asia Pacific). Based on the suggestions from the consultation meetings and internal discussions, an initial set of 34 indicators was identified. A preliminary method of measurement for each of these indicators at the community level and the rationale for their inclusion were also drafted. This exercise was done keeping in mind that the framework will not always be used by research or academic entities but should be user-friendly for local self-governments and community-based organizations.

The refining of the indicator framework and the prioritization exercise (Step 3 and Step 4) was conducted between March 2020 and September 2021.

Step 4: Following this internal exercise, 30 international ABR experts were identified across intergovernmental agencies, academic entities, and civil societies. Twenty of them responded and agreed to assist in the prioritization. The initial set of 34 indicators, the proposed methodology for the data collection for each of these indicators, the rationale for their inclusion, and the methodology for data collection for each one of these indicators were sent over to these experts for prioritization using Google Formsxx over email. The experts were asked to prioritize the indicators based on three different criteria:

1. Appropriateness of the indicator in measuring ABR-specific/sensitive activities at the community level in local communities;
2. Feasibility of measurement in LMIC contexts;
3. Validity of the indicator in detecting changes in response to the intervention on the ground.

The experts were asked to score each indicator from 1 to 5 after carefully assessing the framing, measurement methodology, and reason for inclusion. Experts provided qualitative feedback that was used to draw up criteria for assigning these scores (1–3) to each indicator. In addition to the conceptual framework and the criteria for assigning scores, the data collection methodology drawn up by ReAct Asia Pacific was further refined based on the feedback obtained from the experts. The scores assigned by the experts while evaluating each indicator ranged from 1 to 5. In contrast, each indicator in the framework during data collection were assigned scores of 1 to 3.

Based on the scoring and prioritization given by the experts, 15 indicators were chosen for the final framework. While all indicators were assigned equal weights in the conceptual framework, each indicator can be assigned a minimum score of 1 and a maximum score of 3 depending on the level of progress made by the community in these respective domains.

## 3. Results

Throughout the process, both community stakeholders and experts from different sectors mentioned the need for a framework that can quantify the burden of ABR drivers. The literature review yielded different models of community engagement for ABR, but there were no publications on metrics to quantify ABR drivers or progress made during the 2019–2021 period when this study was carried out. The dialogues with local government suggested the need for a framework that could help identify AMR drivers simultaneously and allocate local resources.

During the consultation meetings in 2019, experts pointed out that the framework should be specifically intended for low resource settings where there are gaps in WASH, access to medicines, and other challenges and take a holistic One Health perspective. The experts suggested that the number of indicators should be manageable for measurement by communities and local government structures. The results of the prioritisation exercise (Step 4) are given in Table 1.

The final set of 15 indicators (see Table 2 below) covered human health, animal health, Agriculture, environment management, and trans-sectoral domains.

As seen from Table 2, the indicators use a ‘One-Health’ approach.

The selection of indicators was based on the scores during the prioritization exercise, no other criterion was applied, and stratification was not carried out. Some of the indicators, such as the ‘over-the-counter’ availability of antibiotics, are specific drivers of the ABR problem in the communities. However, some others, such as the ‘Proportion of households having access to Individual Household Latrine (IHHL) with water supply within the premises of their house’, are linked to systemic capacities to reduce the load of infections in the community and thereby limit the use of antibiotics.

Piloting the indicator framework: The indicator framework was piloted in the community that we were working with to assess its ease of application and feasibility of obtaining information from relevant stakeholder groups. A facilitator from the ReAct Asia Pacific team trained a field worker on the data collection methods using a handbook prepared on data collon. A single trained field worker was employed for data collection after the necessary permissions were obtained from the local self-government body and other concerned institutions.

The piloting of the indicator framework was carried out from October to December 2021 in the selected community in the state of Kerala, India. The ease of application and data availability during the data collection process were optimal. The trained field worker was able to successfully undertake the data collection, and 5% of the collected data were validated through phone calls and in-person visits. In addition, the validity of the data was checked by comparing it with publicly available datasets like the National Family Health Survey. The final results from the piloting process are shown in Table 3.

To test the sensitivity of the indicators to measure change in One Health ABR drivers, targeted context-specific activities were undertaken in the community over a period of six months in collaboration with the community members and local self-government in 2022. A re-assessment that was undertaken following the intervention showed an improvement in the score. The score increased from 34/45 to 38/45. The framework not only aided the research team in considering drawing up an action agenda to address multiple ABR drivers, but it also acted as an entry point for action in the community.

To check the ease of application and validity of the ASC framework, the ASC indicator framework was piloted in four other communities in India in 2022. The four sites were situated in Himachal Pradesh, Bihar, and Assam. The collaborators and local field workers were trained using the standardized data collection handbook. All of these sites successfully piloted the framework, yielding varying scores.

## 4. Discussion

The iterative process to design an indicator framework was based on a shared understanding of the need to engage communities on the ABR issue and to create greater local ownership and sustainable resource mobilization. In the past, there have been attempts in low-resource settings to use performance appraisal frameworks and systematic accountability frameworks to achieve specific programmatic outcomes in the implementation of vertical health programs [21,22]. This approach to mobilize communities has been used in health program implementation in the past with good success [21]. Such measurement frameworks can also provide robust data to funders, program managers, and researchers to assess the real impact of their interventions and help them in prioritizing activities for ABR containment [23]. The authors of this work focused on emulating the success of these approaches/frameworks for ABR, measuring the ‘antibiotic smartness’ of a community through the Antibiotic Smart Communities’ project. Such indicator frameworks can be used for advocacy by comparing the performance of similarly placed regions or local contexts. Since ABR can be considered as an issue with systemic drivers, the containment efforts should be able to reflect the need for systemic changes on the ground [24]. Engaging local communities may be essential for increasing the local ownership of ABR interventions, enhance accountability in implementing machinery, robustly mobilizing resources, and improving the general understanding of the issue [25]. Additionally, it has been demonstrated that community-level behavioral change efforts can be more successful when the relevant local stakeholder groups are fully involved in the efforts [26]. Such a framework which we are proposing can therefore also be a tool for local engagement with the ABR issue and a self-assessment of where the local community stands.

While drafting the methodology of data collection for the indicator framework, the researchers and the experts involved have emphasized the feasibility of collecting data. Therefore, the data collection methodology was made as simple as possible to ensure that trained field workers could collect data in a short duration of time. Some of the piloting data generated using the indicator framework was cross-verified with reports such as the National Family Health Survey 5 (NFHS) [27]. There were no discrepancies between the piloting data and the data gathered through larger and more intensive surveys such as the NFHS. However, the NFHS does not capture data on all indicators in the ASC indicator framework.

One limitation of this indicator framework is that it was developed based on a conceptual framework, which is focused on low-resource settings and not applicable for high-resource settings. The utilization of a consultative process to select and refine the indicators, instead of standard statistical methods, is another limitation. However, the authors have followed the criteria laid down by Statistics New Zealand to select the indicators to overcome the issue of not using statistical techniques (Good Practice Guidelines for Indicator Development and Reporting) [28]. Another limitation was that a cut-off of 15 was chosen, considering that feasibility and other frameworks adopt similar cutoffs and are not analyzed on the basis of scores [28,29].

## 5. Conclusions

The Antibiotic-Smart Communities indicator framework is meant to be a measurement and advocacy tool that can help mobilize local communities in LMICs. An analysis of some of the existing national action plans on antimicrobial resistance has shown gaps in accountability, sustainability, behavioral economics, and local community engagement. This tool can serve to address these gaps, and provide policymakers a way to improve the situation on the ground through appropriate interventions towards optimizing and implementing their national action plans on AMR.

## Figures and Tables

**Table 1:** Scores assigned by the international experts after assessment of each indicator with due consideration of method of measurement and its feasibility, appropriateness, and validity.

Indicator	Total Score Appropriateness (Out of 100)	Total Score Feasibility (Out of 100)	Total Score Validity (Out of 100)	Mean Total Score (Out of 100)
1. Awareness about antibiotic use and antibiotic resistance among general public	77	75	70	74.0
2. Over-the-counter availability of antibiotics in retail pharmacies in the area	85	85	73	81.0 ^#^
3. Proportion of healthcare facilities that have implemented a written Infection Prevention and Control (IPC) plan	65	80	60	68.3
4. Proportion of population using safely managed drinking water services	85	80	82	82.3 ^#^
5. Proportion of healthcare facilities with a written antibiotic protocol for at least three disease/syndrome conditions caused by bacteria	78	80	80	79.3 ^#^
6. Percentage of access antibiotics (as per AWaRe classification of WHO) in total antibiotics dispensed in out-patient settings at healthcare facilities	92	83	83	86.0 ^#^
7. Proportion of healthcare facilities which are accredited by any standard agency (government/private) for quality assurance in delivery of services	77	75	70	74.0
8. Percentage of suspected urinary tract infections (community- or healthcare-associated) being subjected to culture and sensitivity testing	77	67	73	72.3
9. Prevalence of stunting (height for age < −2 standard deviation from the median of the World Health Organization (WHO) Child Growth Standards)	48	67	48	54.3
10. Average under-5 mortality rate (number of deaths among children under 5 years of age compared to number of live births) in the area for the past 3 years	72	83	63	72.6
11. Average out-of-pocket expenditure on healthcare by households in the area	62	68	60	63.3
12. Access to healthcare	70	68	65	67.6
13. Coverage for pediatric vaccines listed in the immunization schedule published by the competent national authority	90	87	88	88.3 ^#^
14. Availability of laboratory services in healthcare facilities within the community	75	78	75	76.0
15. Hygiene facilities in primary and secondary schools in the community	90	87	92	89.6 ^#^
16. Educational initiatives in the past one year to increase awareness about antibiotic or biocide use among farmers	80	80	70	76.6 ^#^
17. Use of highest priority critically important antibiotics in agriculture	88	80	85	84.3 ^#^
18. Regulatory oversight regarding best farm management practices and biosecurity measures	78	78	70	75.3
19. Presence of veterinary health facilities in the community	78	80	75	77.6 ^#^
20. Vaccination coverage for farm animals in the community	82	75	72	76.3
21. Government subsidies or incentives for infrastructural improvement in farms for better infection control practices	70	78	65	71.0
22. Availability of veterinary laboratory services for disease diagnostics	85	83	82	83.3 ^#^
23. Incentive system for farmers who make products without routine use of antibiotics	80	70	73	74.3
24. Presence of schemes to promote local or household-based production of food	63	73	63	66.3
25. Proportion of wastewater treated using any established wastewater treatment technologies, as per WHO’s guidelines on sanitation and health (2019)	80	77	80	79.0 ^#^
26. Biomedical waste management system in healthcare facilities	92	83	82	85.6 ^#^
27. System for disposal of antibiotics and other medicinal waste generated from households	85	65	75	75.0
28. Use of chemical/synthetic pesticides, herbicides, and other biocides in farms	83	72	82	79.0 ^#^
29. Farm waste contaminating water resources in the community	87	70	80	79.0 ^#^
30. Proportion of households having access to Individual Household Latrine (IHHL) with water supply within the premises of their houses	88	87	55	76.6 ^#^
31. Proportion of population covered by at least one social insurance or assurance schemes for health protection	62	70	58	63.3
32. Proportion of population below the nationally accepted poverty line	68	78	65	70.3
33. Proportion of children between ages 5 and 14 receiving nutritional support from government	68	78	68	71.3
34. Female literacy rate	72	77	80	76.3

Legend: ^#^ indicates the final indicator.

**Table 2:** Final list of 15 indicators after prioritization exercise.

1	Hygiene facilities in primary and secondary schools in the community
2	Access to Individual Household Latrine (IHHL) with water supply in households
3	Coverage for pediatric vaccines as per the national immunization schedule
4	Percentage of access antibiotics (as per AWaRe classification of WHO) in total antibiotics dispensed in outpatient settings at healthcare facilities
5	Antibiotic protocols in healthcare facilities
6	Over-the-counter (OTC) availability of antibiotics in retail pharmacies in the area
7	Access to safely managed drinking water services
8	Use of highest priority critically important antibiotics in agriculture
9	Presence of functional veterinary health facilities and services in the community
10	Veterinary laboratory services for disease diagnostics
11	Educational initiatives on antibiotic use among farmers
12	Biomedical waste management system in healthcare facilities
13	Treatment of wastewater generated in households
14	Use of chemical/synthetic pesticides, herbicides, and other biocides in farms
15	Farm waste contaminating water resources in the community

**Table 3:** Results of the piloting of the indicator framework carried out in a selected community in India.

Indicator	Performance of the Community			Score
Hygiene facilities in primary and secondary schools in the community	Good	Reasonable	Inadequate	3
Access to Individual Household Latrine (IHHL) with water supply in households	All	Most	Some	3
Coverage for pediatric vaccines as per the national immunization schedule	High	Reasonable	Low	3
Percentage of access antibiotics (as per AWaRe classification of WHO) in total antibiotics dispensed in outpatient settings at healthcare facilities	High	Reasonable	Low	2
Antibiotic protocols in healthcare facilities	All	Some	None	2
Over-the-counter (OTC) availability of antibiotics in retail pharmacies in the area	Poor OTC availability	Partial OTC availability	Free OTC availability	1
Access to safely managed drinking water services	All	Most	Some	3
Use of highest priority critically important antibiotics in agriculture	None	Some	High	2
Presence of functional veterinary services, health facilities, and services in the community	Fully functional	Semi-functional	Not functional	3
Veterinary laboratory services for disease diagnostics	Fully functional	Semi-functional	Not functional	2
Educational initiatives on antibiotic use among farmers	Fully functional	Semi-functional	Not functional	1
Biomedical waste management system in healthcare facilities	All	Some	None	2
Treatment of wastewater generated in households	All	Most	Some	2
Use of chemical/synthetic pesticides, herbicides, and other biocides in farms	Low	Significant	High	2
Farm waste contaminating water resources in the community	High	Some	None	3
Final score				34/45

## Data Availability

The handbook for data collection will be published online on the ReAct tool box. ReAct Toolbox for Action on Antibiotic Resistance. Available online: https://www.reactgroup.org/toolbox/ (accessed on 28 December 2023).
